# Novel Coronavirus (COVID-19) Infection-Attributed Acute Pancreatitis: A Case Report and Literature Review

**DOI:** 10.7759/cureus.15725

**Published:** 2021-06-17

**Authors:** Rohit Chandra, Nicholas J Lazar, Seth Goldman, Zaid Imam, Ramy Mansour

**Affiliations:** 1 Internal Medicine, Beaumont Health, Royal Oak, USA; 2 Gastroenterology, Beaumont Medical Center, Royal Oak, USA; 3 Gastroenterology, Endoscopic Solutions PC, Clarkston, USA

**Keywords:** acute pancreatitis, covid-19, viral pancreatitis, atlanta classification, sars-cov-2

## Abstract

Novel coronavirus (COVID-19) has spread widely across the world inducing a global health crisis. Predominant signs of infection involve respiratory symptoms such as cough and dyspnea. Investigation into COVID-19 infection-associated gastrointestinal symptoms remains fluid. COVID-19-induced acute pancreatitis has been recorded from greater than 20 countries at this time. Herein, we submit a case of COVID-19-attributed acute pancreatitis, as well as a comprehensive assessment of previously reported cases of COVID-19-attributed acute pancreatitis.

## Introduction

Gastrointestinal manifestations of the novel coronavirus (SARS-CoV-2) are common with 53% of patients presenting with coronavirus infections (COVID-19) complaining of at least one gastrointestinal complaint, and 35% of patients presenting with mild elevations of aminotransferases and hyperbilirubinemia [1]. Data on pancreatic involvement in patients with COVID-19 is limited. A small cohort of 71 patients reported hyperlipasemia in nine patients and no cases of acute pancreatitis (AP). The hyperlipasemia in this cohort was attributed to subclinical pancreatic involvement or non-pancreatic hyperlipasemia [2]. We hereby report a case of COVID-19-attributed acute pancreatitis encountered on a review of hospitalized 1305 patients with COVID-19 and summarize the clinical presentation and outcomes of 26 other reported cases in the literature [3-25].

## Case presentation

A 53-year-old African American male presented in March 2020 with one week of progressively worsening shortness of breath and mild confusion. He also reported generalized abdominal pain for three days. His medical history included hypertension, benign prostatic hyperplasia, hyperlipidemia and bronchial asthma. His medications were triamterene, amlodipine, tamsulosin, atorvastatin and inhaled fluticasone. He had no known history of diabetes mellitus or pancreatitis. He denied alcohol or tobacco use. He appeared with Kussmaul breathing, a respiratory rate of 40 breaths/minute, and an initial oxygen saturation of 99% on 3 liters of oxygen by nasal cannula.

**Figure 1:**
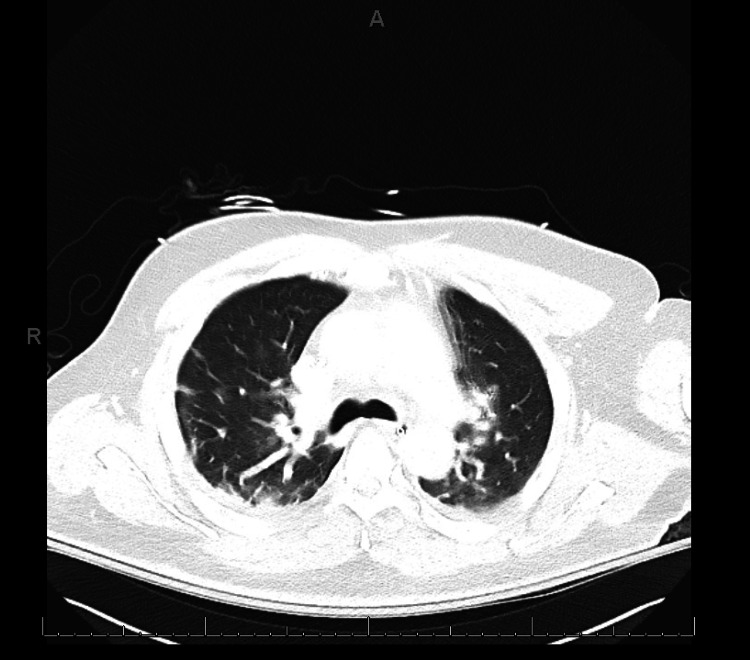
Computed tomography of chest

**Figure 2:**
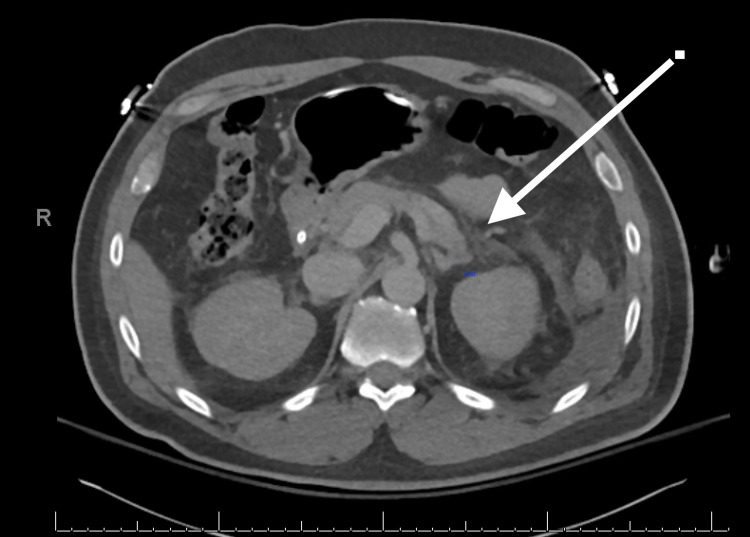
Computed tomography of the abdomen

Initial laboratory workup summarized in Table 1 demonstrated neutrophilic leukocytosis, severe hyperglycemia, high anion-gap metabolic acidosis, hyperlipasemia to 1200 U/L and acute kidney injury. Liver aminotransferases, and serum bilirubin were normal. A serum alcohol level was undetectable. These findings confirmed the diagnosis of severe diabetic ketoacidosis. A chest X-ray demonstrated bibasilar patchy infiltrates and a SARS-CoV-2 reverse transcriptase polymerase chain reaction (RT-PCR) was positive on a nasopharyngeal swab. The patient was intubated for respiratory failure in the setting of severe metabolic acidosis. High resolution computed tomography (CT) of the chest showed diffuse bibasilar atelectasis and patchy infiltrative changes (Figure 1). CT of the abdomen and pelvis demonstrated acute pancreatitis affecting the head and tail of the pancreas with no peripancreatic fluid collections or necrosis (Balthazar Grade C), with a normal gallbladder and biliary tract. Specifically, imaging showed peripancreatic fatty infiltrative changes that is seen about the head and tail of the pancreas (Figure 2). Aggressive fluid resuscitation, and intravenous insulin infusion were initiated. The patient received plaquenil, vitamin C, and zinc for five days when ventilated allowing for extubation after receiving a total of 13 days of mechanical ventilation. Renal replacement therapy with continuous veno-venous hemodialysis was required for 20 days with subsequent renal recovery. Inflammatory markers were tested at admission and peaked on day 6 of admission and subsequently downtrended during admission. He subsequently recovered and was discharged after 30 days of hospitalization to a rehabilitation facility. On follow-up four months from hospitalization, he is doing well having recovered completely with no residual renal impairment, respiratory failure, and remains on insulin for diabetes.

**Table 1:** Summary of laboratory values on initial presentation

Laboratory Test	Value	Lab normal	Units
A. Complete Blood Count			
Total White blood cell count	20,300	3,500-10,100	Cells/mm^3^
Neutrophil count	16,100	1,600-7,200	Cells/mm^3^
Lymphocyte Count	1,400	1,100-4,000	Cells/mm^3^
Platelet Count	237,000	150,000-400,000	Cells/mm^3^
Serum hemoglobin	15.5	13.5-17.0	g/dL
B. Serum chemistries			
Albumin	3.7	3.5-5.1	g/dL
Sodium	132	135-145	mEq/L
Potassium	5.9	3.5-5.2	mEq/L
Chloride	99	98-111	mEq/L
Bicarbonate	7	20-29	mEq/L
Serum glucose	1291	60-99	mg/dL
Blood urea nitrogen	89	7-25	mg/dL
Creatinine	5.44	0.60-1.30	mg/dL
Lactic acid	10.4	0.5-2.0	mg/dL
Beta hydroxybutyric acid	11.92	0.02-0.27	mmol/L
Aspartate aminotransferase (AST)	26	<35	U/L
Alanine aminotransferase (ALT)	29	9-47	U/L
Total bilirubin	0.3	0.3-1.2	mg/dL
Alcohol level	Undetectable	<10	mg/dL
Lipase level	1200	0-60	U/L
Triglyceride level	677	0-149	mg/dL
C. Arterial blood gas			
Arterial pH	7.11	-	-
Serum pCO2	11	-	mmHg
Serum pO2	109	-	mmHg
D. Urine drug screen	Negative	-	-
E. C-reactive protein (CRP)	51.2		

## Discussion

The novel coronavirus infection has been diagnosed in 95 million people and over 2 million deaths have been publicly recorded, and while its respiratory symptoms predominate clinical presentations, much has yet to be elucidated about its gastrointestinal complications including acute pancreatitis (AP). Initial cases of COVID-19-attributed AP were reported as early as June 2020 by Miao et al. [25]. Viral affinity to the pancreatic ACE-2 receptors and their activation have been proposed as a possible pathophysiological mechanism for COVID-19-attributed AP [2,26]. Other mechanisms including direct intra-acinar cellular replication similar to that resulting from hepatitis viruses are also possible [27].

Virus-attributed AP has been defined as the occurrence of AP as defined by the Revised Atlanta Classification (RAC), a temporal association with the implicated viral infection, and an exclusion of common etiologies of AP (biliary, alcohol, medication-induced, and metabolic causes) [27]. This definition is met by our reported case attributing AP to COVID-19. Organ failures (pulmonary and renal failure) occurring during the patient’s admission appear more related to the severity of his diabetic ketoacidosis and acute respiratory distress syndrome (ARDS) resultant from the critical COVID-19 infection rather than the severity of his interstitial acute pancreatitis episode. Credence needs to be given to whether the reported patient could have developed AP secondary to diabetic ketoacidosis given prior reports on the association [28]. However, these associated cases typically exhibit severe hypertriglyceridemia greater than 1000 mg/dL or some degree of alcohol use, not seen in the reported patient [28-30].

To better discern the relationship between AP and COVID-19, we performed a review of PubMed and EMBASE databases for any published cases of COVID-19-attributed AP in adults since December 2019 as defined by their reporting authors. We then analyzed these cases to identify whether they met the definition of virus-attributed AP by Imam et al., in addition to the patients’ clinical characteristics and outcomes [27].

A total of 26 cases were identified from 15 different countries with a median presenting age of 49.6 years [3-26]. Individual case characteristics are summarized in Table 2. Of published reports, all cases were temporally associated with COVID-19 infections and all cases met RAC definitions for AP diagnosis. Attempting to exclude other etiologies of acute pancreatitis, all but one case excluded alcohol use as an etiology, only 21 (80.8%) patients had radiologic studies to exclude cholelithiasis, 15 (58%) cases reported on evaluation for hypercalcemia, 19 (73%) cases on excluding hypertriglyceridemia, and 16 (62%) excluded drug-induced acute pancreatitis. This unfortunately introduces bias in published literature on the topic. An evaluation of the methodological quality of the case reports utilizing the Murad tool is summarized in Table 3.

**Table 2:** Individual cases of COVID-19-attributed acute pancreatitis published in the literature HyperTG: Hypertriglyceridemia

Author Name	Age (YRS)/Sex	Necrosis	Severity	Outcome	F/U	EtOH	Gallbladder Disease	Hypercalcemia	Medication Use	HyperTG
Aloysius et al. [3]	36/F	N	Severe	Recovery	14	N	N	-	N	N
Kataria et al. [4]	49/F	N	Severe	Recovery	7	N	N	N	N	N
Mazrouei et al. [15]	24/M	N	Mild	Recovery	3	N	N	-	N	-
Anand et al. [19]	59/F	N	Mild	Recovery	7	N	Cholecystectomy	-	N	N
Kumaran et al. [20]	67/F	Y	Severe	Recovery	10	N	N	N	N	N
Meireles et al. [21]	36/F	N	Mild	Recovery	3	N	N	N	N	N
Cheung et al. [22]	38/M	N	Mild	Recovery	0	N	N	N	N	N
Hadi et al. (1) [23]	47/F	N	Severe	Recovery	0	N	N	-	-	N
Hadi et al. (2) [23]	68/F	N	Severe	Recovery	22	N	-	N	-	N
Hadi et al. (3) [23]	71/M	N	Severe	Death	0	N	-	-	-	-
Rabice et al. [24]	36/F	N	Moderate Severe	Recovery	9	N	N	N	N	N
Brikman et al. [5]	61/M	N	Mild	Recovery	2	N	N	N	N	N
Bokhari and Mahmood [6]	32/M	N	Mild	Recovery	3	N	N	N	-	N
Gonzalo-Voltas et al. [7]	76/F	N	Mild	Recovery	5	N	N	-	-	N
Gadiparthi et al. [8]	40/M	N	Severe	Recovery	6	N	N	-	-	Y
Karimzadeh et al. [9]	65/F	N	Mild	Recovery	18	N	-	-	-	-
Wang et al. (1) [18]	42/M	N	Severe	Mortality	10	N	N	N	N	N
Wang et al. (2) [18]	35/M	N	Mild	Recovery	18	-	N	N	N	N
Lakshmanan and Malik [10]	68/M	N	Mild	Recovery	7	N	N	N	N	N
Kurihara et al. [11]	55/M	N	Severe	Recovery	26	N	N	N	N	N
Dietrich et al. [12]	72/M	N	Mild	Recovery	59	N	N	-	-	-
Alves et al. [13]	56/F	N	Severe	Recovery	35	N	N	N	-	N
Patnaik et al. [14]	29/M	N	Mild	Recovery	29	N	N	-	-	N
Purayil et al. [16]	58/M	N	Mild	Recovery	4	N	N	-	N	-
Miao et al. [25]	26/F	N	Mild	Recovery	7	N	-	N	N	N
Kandasamy [17]	45/F	N	Mild	Recovery	7	N	N	N	N	-

**Table 3:** Methodological quality assessment for included publications using the Murad tool (1) Did the patient(s) represent the whole case(s) of the medical center? (2) Was the diagnosis correctly made? (3) Were other important diagnoses excluded? (4) Were all important data cited in the report? (5) Was the outcome correctly ascertained? * Meeting all five criteria renders the publication at low risk of bias, meeting four criteria at moderate risk of bias, and meeting three criteria at high risk of bias.

Author Name	N	Year	Q1 ^(1)^		Q2 ^(2)^		Q3 ^(3)^		Q4 ^(4)^		Q5 ^(5)^		Risk of Bias
			Y	N	Y	N	Y	N	Y	N	Y	N	
Aloysius et al. [3]	1	2020	Y		Y		Y		Y		Y		Low
Kataria et al. [4]	1	2020	Y		Y		Y		Y		Y		Low
Mazrouei et al. [15]	1	2020	Y		Y		Y		Y		Y		Low
Anand et al. [19]	1	2020	Y		Y		Y		Y		Y		Low
Kumaran et al. [20]	1	2020	Y		Y		Y		Y		Y		Low
Meireles et al. [21]	1	2020	Y		Y		Y		Y		Y		Low
Cheung et al. [22]	1	2020	Y		Y		Y		Y		Y		Low
Hadi et al. [23]	3	2020	Y		Y			N	Y		Y		Moderate
Rabice et al. [24]	1	2020	Y		Y		Y		Y		Y		Low
Brikman et al. [5]	1	2020	Y		Y		Y		Y		Y		Low
Bokhari and Mahmood [6]	1	2020	Y		Y		Y		Y		Y		Low
Gonzalo-Voltas et al. [7]	1	2020	Y		Y		Y		Y		Y		Low
Gadiparthi et al. [8]	1	2020	Y		Y			N	Y		Y		Moderate
Karimzadeh et al. [9]	1	2020	Y		Y			N	Y		Y		Moderate
Wang et al. [18]	2	2020	Y		Y		Y		Y		Y		Low
Lakshmanan and Malik [10]	1	2020	Y		Y		Y		Y		Y		Low
Kurihara et al. [11]	1	2020	Y		Y		Y		Y		Y		Low
Dietrich et al. [12]	1	2020	Y		Y			N	Y		Y		Moderate
Alves et al. [13]	1	2020	Y		Y		Y		Y		Y		Low
Patnaik et al. [14]	1	2020	Y		Y		Y		Y		Y		Low
Purayil et al. [16]	1	2020	Y		Y		Y		Y		Y		Low
Miao et al. [25]	1	2020	Y		Y		Y		Y		Y		Low
Kandasamy [17]	1	2020	Y		Y		Y		Y		Y		Low

In terms of outcomes, mean length of follow-up was 11 ± 13 days and only one case of necrotizing pancreatitis was reported. Mortality occurred in two patients attributed to respiratory failure in both cases [18,23], while the remainder of patients survived their admission. Severity classification by RAC definitions was performed, identifying 10 (38.4%) cases of severe AP, one (3.8%) case of moderately severe AP, and 15 (57.7%) cases of mild AP. It is worthy to note given the high prevalence of organ failures in critical COVID-19 infections, these severity definitions are likely skewed by the severity of the primary infection (COVID-19) rather than the AP episode. Prior reports on hepatitis A and E-associated AP identified that outcomes were largely dependent on the occurrence of acute liver failure rather than the severity of the AP episode, suggesting the role of pancreatic involvement as a bystander organ [31,32]. This likely can be extrapolated in the setting of COVID-19-attributed AP as well.

## Conclusions

With the ongoing COVID-19 pandemic, patients are presenting at emergency departments with a litany of different complaints. COVID-19-associated AP is infrequent and its role in affecting patient outcomes is unclear. Further investigation is needed to elucidate whether pancreatic involvement affects patient outcomes, and how best to manage these patients as aggressive resuscitation in patients with critical COVID-19 may worsen concurrent pulmonary pathology.
